# Multiparametric Monitoring in Equatorian Tomato Greenhouses (II): Energy Consumption Dynamics

**DOI:** 10.3390/s18082556

**Published:** 2018-08-04

**Authors:** Mayra Erazo-Rodas, Mary Sandoval-Moreno, Sergio Muñoz-Romero, Mónica Huerta, David Rivas-Lalaleo, José Luis Rojo-Álvarez

**Affiliations:** 1 Departamento de Eléctrica y Electrónica, Universidad de las Fuerzas Armadas ESPE, Av. General Rumiñahui s/n, Sangolquí 171-5-231B, Ecuador; drrivas@espe.edu.ec; 2 Departamento de Teoría de la Señal y Comunicaciones, Sistemas Telemáticos y Computación, Universidad Rey Juan Carlos, 28943 Fuenlabrada, Spain; sergio.munoz@urjc.es (S.M.-R.); joseluis.rojo@urjc.es (J.L.R.-Á.); 3 Departamento de Ciencias Exactas, Universidad de las Fuerzas Armadas ESPE, Av. General Rumiñahui s/n, Sangolquí 171-5-231B, Ecuador; mjsandoval@espe.edu.ec; 4 Center for Computational Simulation, Universidad Politécnica de Madrid, Boadilla del Monte, 28660 Madrid, Spain; 5 Carrera de Telecomunicaciones, Universidad Politécnica Salesiana, 010105 Cuenca, Ecuador; mhuerta@ups.edu.ec

**Keywords:** wireless sensor networks, stochastic process, energy consumption, tomato greenhouse, M-mode plots.

## Abstract

Tomato greenhouses are a crucial element in the Equadorian economy. Wireless sensor networks (WSNs) have received much attention in recent years in specialized applications such as precision farming. The energy consumption in WSNs is relevant nowadays for their adequate operation, and attention is being paid to analyzing the affecting factors, energy optimization techniques working on the network hardware or software, and characterizing the consumption in the nodes (especially in the ZigBee standard). However, limited information exists on the analysis of the consumption dynamics in each node, across different network technologies and communication topologies, or on the incidence of data transmission speed. The present study aims to provide a detailed analysis of the dynamics of the energy consumption for tomato greenhouse monitoring in Ecuador, in three types of WSNs, namely, ZigBee with star topology, ZigBee with mesh topology (referred to here as DigiMesh), and WiFi with access point topology. The networks were installed and maintained in operation with a line of sight between nodes and a 2-m length, whereas the energy consumption measurements of each node were acquired and stored in the laboratory. Each experiment was repeated ten times, and consumption measurements were taken every ten milliseconds at a rate of fifty thousand samples for each realization. The dynamics were scrutinized by analyzing the recorded time series using stochastic-process analysis methods, including amplitude probability functions and temporal autocorrelation, as well as bootstrap resampling techniques and representations of various embodiments with the so-called M-mode plots. Our results show that the energy consumption of each network strongly depends on the type of sensors installed in the nodes and on the network topology. Specifically, the CO${}_{2}$ sensor has the highest power consumption because its chemical composition requires preheating to start logging measurements. The ZigBee network is more efficient in energy saving independently of the transmission rate, since the communication modules have lower average consumption in data transmission, in contrast to the DigiMesh network, whose consumption is high due to its topology. Results also show that the average energy consumption in WiFi networks is the highest, given that the coordinator node is a Meshlium™ router with larger energy demand. The transmission duration in the ZigBee network is lower than in the other two networks. In conclusion, the ZigBee network with star topology is the most energy-suitable one when designing wireless monitoring systems in greenhouses. The proposed methodology for consumption dynamics analysis in tomato greenhouse WSNs can be applied to other scenarios where the practical choice of an energy-efficient network is necessary due to energy constrains in the sensor and coordinator nodes.

## 1. Introduction

The tomato, technically known as *Solanum lycopersicun*, is one of the most significant products contributing to the economy of the Ecuadorian agricultural sector [1]. For this reason, tomatoes are usually grown under greenhouses to increase their production. This structured environment facilitates the implementation of internal and external climate monitoring systems. On the other hand, wireless sensor networks (WSNs) are currently the telecommunication systems with the highest penetration in the use of the population for specialized applications such as precision agriculture. WSNs are implemented with autonomous sensor nodes that collect data packages and transmit them to a coordinator at the expense of an external energy source, such as batteries [2,3,4]. Therefore, the energy consumption of the nodes is an especially relevant parameter for the operation quality of these networks.

At present, the energy consumption characterization of WSNs nodes in terms of telecommunication infrastructure is a topic of interest, because it affects about 1% of the world’s electricity consumption, and it has been exponentially increasing [5,6]. The scientific and technical literature includes works focused to the analysis of energy consumption in relation to the operation mode [7] and the performance of the operating system [8] in the communication radios. In addition, energy-saving techniques using hardware [9,10,11,12] and software [13] have been considered, and most of these studies are applied to WSNs with ZigBee technology and star topology. To our best knowledge, few works involve an extensive knowledge of the detailed statistical description of consumption dynamics and its ergodic properties, as well as a rigorous analysis of the estimation of stationarity. In addition, the contributions with respect to comparisons of the energy consumption behavior of WSNs for different wireless technologies, network topologies, and transmission rates are scarce.

Based on this background, the present study aims to provide a detailed analysis of the dynamics of the energy consumption in three types of WSNs, namely, ZigBee with star topology, ZigBee with mesh topology (so-called here DigiMesh), and WiFi with access point topology for tomato greenhouse monitoring in Ecuador. We want to provide an accurate statistical characterization of the behavior of WSNs, relating energy consumption, transmission rate, and network topology to provide researchers with the selection criteria of the appropriate network design in terms of energy savings, and oriented to monitoring the climate of greenhouses. For this purpose, the the electrical current time series recorded at each node were studied using stochastic-process analysis methods, including amplitude probability functions and temporal autocorrelation estimation. We also used bootstrap resampling techniques to provide with non-parametric statistical description of confidence intervals (CIs) when convenient, as well as the so-called M-mode plots to represent and compare the statistical properties of the several realizations. The two companion papers are devoted to the analysis of the WSN topologies and their configuration and to the study of the dynamics of the environmental measurements, both of them in Equadorian tomato greenhouse monitoring [14,15]. The first one has the detailed description of the complete system and network setup, and the second one has a short summary of the proposed system for tomato greenhouse monitoring.

This paper is organized as follows. Section 2 reviews some documents related with the energy consumption dynamics in WSNs. Section 3 describes the statistical analysis tools that are used to study the dynamics of the energy consumption variables. Section 4 presents the detailed results when analyzing each WSN at different speeds. Finally, Section 5 gives the discussion and conclusions.

## 2. Related Work

WSN is a modern technology that has been widely used in a variety of applications, such as greenhouse parameter control in precision agriculture [16,17], quality water monitoring systems [18,19], military applications [20], intelligent street lighting systems [21], air pollution monitoring systems [22], urban traffic characterization [23], oil industry [24], or health status monitoring [25], among many others. Today, WSNs offer some advantages over traditional wireless communication systems, such as low cost, ease of installation, real time, reliability, and connectivity [26]. Although the choice of WSN technology is attractive, there are still some limitations present, such as energy consumption, low battery life, redundant data acquisition, or low duty cycle.

One of the today problems in the development of WSN systems comes from the high energy consumption of the sensor nodes, due to its direct impact on the WSN useful life [27]. According to the literature [28,29], the relevant factors for energy consumption are inside the sensing module (active sensors, sampling rate, and sensor type), inside the processing module (active state, clock frequency, and microcontroller), and inside the communication module (bandwidth, radio, distance, duty cycle, and topology). The heaviest energy consumer is the communication module used for the transmission and reception of data among sensor nodes, and the most energy-demanding elements are at the radio subsystems. Batteries are usually constrained to be small and they are in trouble when required to support long-distance communications due to transmitted power constraints [7].

Table 1 shows a compilation of research works on the evaluation of WSNs topologies, transmission reliability, energy-consumption optimization techniques, and energy modeling of sensor nodes. In summary, it can be seen that the research is often limited with respect to the analysis of the energy consumption dynamics of the sensor nodes, with the simultaneous consideration of different communication technologies and topologies, in addition to the incidence of the data transmission speed. However, and to our best knowledge, a detailed analysis on the consumption dynamics in terms of detailed statistical descriptions of the consumption time series evolution is missing in the literature.

## 3. Statistical Analysis of the Consumption Dynamics

In this section, we briefly describe the data analysis tools that have been used to study the dynamics of a set of energy consumption variables. From a time-process analysis viewpoint, we used methods allowing us to scrutinize the statistical nature of the recorded signals (histograms and autocorrelation) and their stationarity (autocorrelation and M-mode representations). To provide a detailed statistical description, nonparametric statistics (in terms of bootstrap resampling techniques) were used to calculate CIs when convenient [49]. We describe in Appendix A the theoretical fundamentals for this set of statistical analysis tools, and the interested reader is encouraged to find therein a more detailed mathematical notation and statistical description for all these elements.

In addition to the conventional time representation of signal and time processes, in this work, we often use the representation of the observed process using a bidomain support. For this purpose, we introduce here the so-called M-mode representation, which is just a bidomain plot of time signals (or its estimated statistical descriptors) accounting for a second and convenient representation domain. This M-mode representation has been recently used in cardiac signal processing to provide with spatial-temporal representations of a set of time signals along a line of spatial sampling points [50,51]. This M-mode representation can be advantageously used not only for signals, but also for their statistical descriptions. In our case, it is also applied to the characterization of the energy consumption time processes in terms of their probability distributions and of their autocorrelation, as described next.

The probability density function (often denoted as *pdf*) describes the relative probability that the random variable falls in a specific region of the probability space, and it is defined by the integral of the density of this variable between the limits of said region. Note that, since the *pdf* representation on a linear scale can limit the visual perception of small yet relevant values, especially in the tails, we also considered here its representation in log-log scale when convenient. The M-mode plots were also used to represent jointly the estimated *pdf* of each realization to quantitatively and visually analyze the similarities among them. In this work, the *pdf* was estimated in terms of the normalized histogram, either for a given realization, or for the complete process. The histogram is the representation in the form of bars of the distribution of a set of data from a given variable, so that each bar has equal width *a* and the bar heights are proportional to the relative frequency of the data represented in that interval or bin [52]. In our application, this simple technique offers a panoramic and ordered view of the estimated *pdf* of the consumption in each node of the three networks to easily identify the trends, variability, and shape of the energy consumption distributions.

In addition to the estimated averaged *pdf*, it is informative to define a CI for it. However, this calculation is not simple, because the underlying distributions are not known in practice. For this reason, a non-parametric method is required to estimate the density of the estimated statistics, which is required to be robust with respect to the underlying distribution shape. Bootstrap resampling is a statistical estimation method which has been widely applied in Probability Theory and in Statistical Inference for the construction and estimation of CI of a wide variety of statistical processes. This technique uses the data collected from a given process and the plug-in principle to randomly generate new simulated samples (resamples) of the same size as the original sample [49]. Bootstrap resampling techniques are widely used in practical problems; they are versatile and robust with respect to the underlying statistical distribution; and they can be applied to virtually any estimator that can be obtained computationally [53].

The simple autocorrelation function (SAF) is a statistical tool that determines the correlation or similarity between a signal and its out-phase replica as a function of delay time (commonly denoted as τ) [54]. In the field of signal processing, the autocorrelation facilitates the analysis of seasonality of functions or time series in the time domain, especially when focused on applications related with recognition of repetitive statistical patterns, on detection of (near-)periodic signals masked by noise, or on the study of autoregressive moving average processes, among many others [55].

## 4. Experiments and Results

In this section, we first describe the configuration parameters and the connections of the measurement equipment used in the experimental stage to acquire the energy consumption of each node of the three networks. The data collection experiments were repeated ten times, all under the same scenario, to characterize the statistical behavior of the energy consumption of each element of the networks. Next, we present the dynamics of the consumption signals that were analyzed with custom-developed software in Matlab™. Selected and representative graphs of time series, probability distributions, and SAF, were studied in each node and at different transmission rates. The scheme of the comparative study was the following. Initially, we defined some relevant characteristics of the different graphical representations of the DigiMesh network, as given by its trend, mean, standard deviation, probability distribution, and seasonality, at a fixed and low rate of 9600 bauds. Next, we compared these signals with other transmission speeds, and we identified the significant peculiarities and got the corresponding conclusions. The same procedure was applied to the ZigBee and WiFi networks. We end with a global analysis of the results of the three networks to discern the incidence of communication technology and the transmission rate with respect to the battery consumption of each kind of node.

### 4.1. Setup of Energy Consumption Measurements

**Figure 1:**
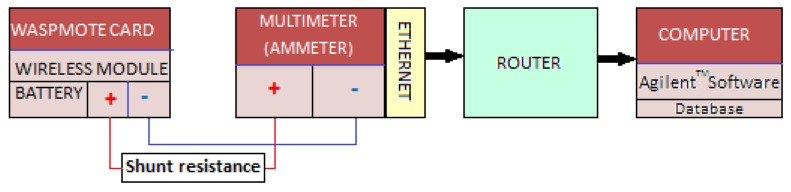
Acquisition and storage of the current consumption in each node.

The efficient energy consumption in the nodes of WSNs is relevant for their performance, because it increases the operating time of the batteries and reduces the risk of data loss due to lack of energy in a network node. In this research, the energy consumption measurements of each node were acquired and stored in the laboratory according to the scheme in Figure 1. An experiment was developed for each network, and in all cases the link between the nodes was with line of sight and 2-m length. The battery of each node was connected to the Agilent™ 34410A multimeters for the current consumption measurements, and the recordings were transmitted through a local area network to the computer. The multimeter was configured from the computer using SCPI commands, and current data were transferred to a Microsoft Excel™ database with Agilent IntuiLink™ software, where data were subsequently processed and analyzed with custom-made Matlab™ functions.

The relevant aspects that were considered for the experimentation phase of the three networks are shown in Table 2. Baud rates were selected according to the technical specifications given by the manufacturer of the Waspmote processing card and the communication modules. The parameters configured on multimeters were static IP address, DHCP protocol disabled, and sample count to fifty thousand. The resolution of the device was also analyzed according to the integration time, which was defined as the period during which the analog-to-digital converter in the multimeter sampled the input signal measurements. This time is typically expressed in number of power line cycles (NPLC) [56]. The integration time is directly related to the resolution of the device and inversely related to the noise and measurement speed. For the first experiment, a low NPLC was selected for the three networksto have a moderate acquisition time of each measurement. However, we observed that the noise in the resulting signals was considerable, especially in the DigiMesh and WiFi networks. The NPLC values were configured differently for each network after the noise level analysis, and shown in Table 2. For the three networks, the experiment was repeated ten times in the same scenario, and fifty thousand samples were acquired every ten milliseconds to statistically verify the similarity of the signals and to determine the behavior pattern of the energy consumption of each node at different speeds.

### 4.2. Energy Consumption Dynamics of DigiMesh Network

In Appendix B, we present the detailed analysis with all the proposed elements for the Digimesh Network when working at 9600 bauds. This complete description is only detailed for that case, so that the interested reader can better follow the scope and contribution of every kind of analysis addressed here. Based on the methodology described in detail in Appendix B, we present in this section only the relevant results of the comparative study for the energy consumption dynamics of the DigiMesh network at 9600, 57,600 and 19,200 baud rates.

**Figure 2:**
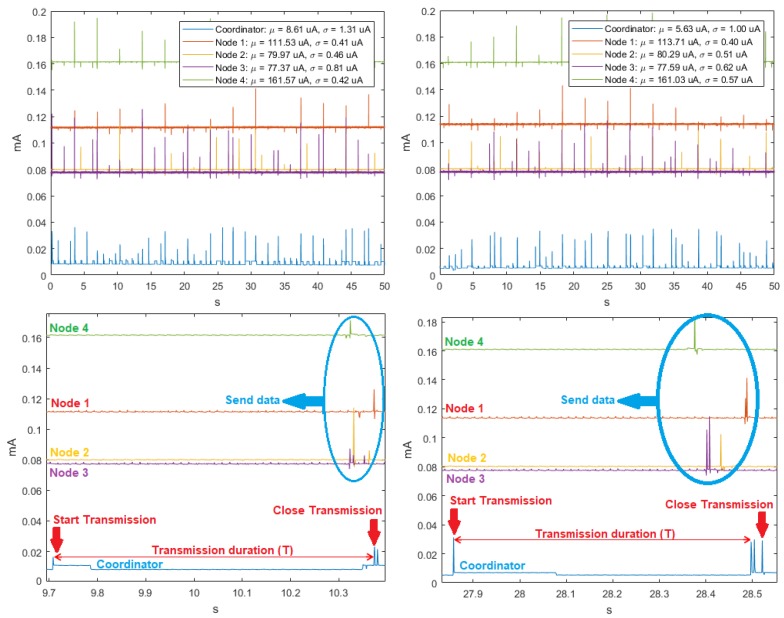
Time signals for DigiMesh network in two example realizations: (**Left**) at 19,200 bauds; (**Right**) at 57,600 bauds; (**Top**) complete time signals; and (**Bottom**) signal sections with closer detail.

For three transmission rates, the time signals were homoscedastic, as seen in the example in Figure 2, and also the values of μ and σ of the sensor nodes and the peak-transmission sequences were similar. In addition, we verified that regardless of the transmission speed, the average power consumption was the highest for Node 4 and the lowest for Node 3. In the particular case of the Coordinator Node, we observed that, when the velocity increased, the averaged of energy consumption decreased. Table 3 summarizes the average consumption, its deviation, and the transmission duration of each realization for the three analyzed baud rates. The standard deviations were low, being below 1 μA at the Sensor Nodes and below 1.2 μA at the Coordinator Nodes. The transmission duration for the three speeds was about 0.68 s. This value was determined by first taking the mode of the samples in each realization, and then averaging them for all the realizations.

**Figure 3:**
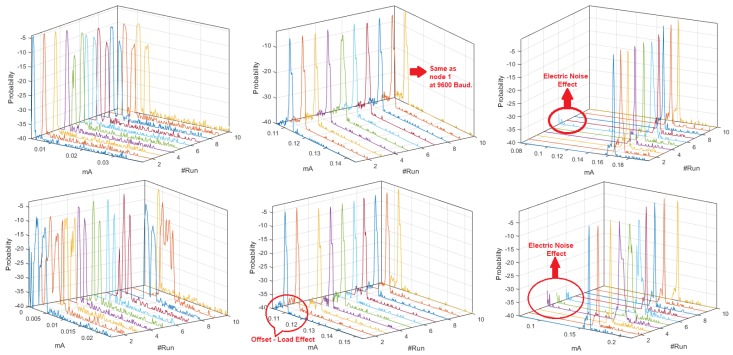
M-mode of *pdf* with distinctive characteristics for DigiMesh network. From left to right: Coordinator Node, Node 1, and Node 4. (**Top**) At 19,200 bauds; and (**Bottom**) at 57,600 bauds.

Figure 3 shows the set of *pdf*, whose behavior was different from the signals analyzed in Appendix B. The distributions of the ten realizations for the Coordinator Node at 19,200-baud rate showed more stable behavior than those obtained at 9600 and 57,600 bauds. However, the load effect remained present in all runs of the three speeds, which was evidenced in the progressive phase shift of the signals. The plots of Node 1 at 9600 and 19,200 bauds were similar, while a slight offset of the two initial runs was observed at 57,600 bauds. This was caused by the low charge in the battery. We conclude that the load effect on the sensor nodes is hardly visible at high transmission speeds. Based on the high likeness of the *pdf* of Nodes 2 and 3 for the three speeds, the modification of the value of this parameter does not significantly influence the energy consumption. The offset that was observed in Node 4 *pdf* at 9600 bauds due to impedance decoupling was imperceptible for these other speeds. Nevertheless, in some realizations, the increase in the transmission speed caused the presence of low consumption values which were distant from the central distribution curve, and these values corresponded to electrical noise, which was more noticeable at 57,600 bauds.

**Figure 4:**
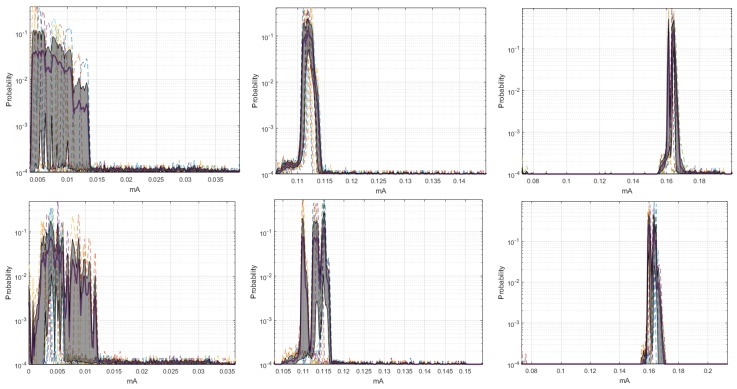
CI of *pdf* with distinctive characteristics for DigiMesh network. From left to right: Coordinator Node, Node 1, and Node 4. (**Top**) At 19,200 bauds; and (**Bottom**) at 57,600 bauds.

By means of the graphs of the CI of each network element at 19,200 and 57,600 bauds, we can identify some characteristics with respect to the 9600 bauds, and the relevant signals are shown in Figure 4. The Coordinator Node was the element with the widest CI for the three speeds; therefore, the variation between the realizations because of the load effect was present in all cases, regardless the communication transmission rate. The multimodal distribution and the size of the intervals were similar at the speeds of 9600 and 57,600 bauds, however, the variation between modes at 19,200 bauds was visibly softer and the CI width was larger. Based on this, we deduced that the data dispersion was more stable, but the loading effect was slightly larger. In addition, the range with the highest concentration of data consumption for this node decreases with increasing transmission speed. The change in the transmission rate for Nodes 2 and 3 did not influence the behavior of energy consumption, since the *pdf*, the margin of highest data concentration, and the narrow width of the CI were very similar at the three speeds. In contrast, the similarities of these parameters for Sensor Node 1 were evident at 9600 and 19,200 bauds, but the *pdf* was multimodal, and the CI increased considerably at 57,600 bauds, due to the lag between realizations that was previously explained in the M-mode analysis of this node. The increase in the transmission speed in Node 4 did not significantly change the consumption range with higher data concentration, however, the number of modes of the probability distribution was reduced from three to two, and the CIs were reduced in both speeds, with the narrowest one being obtained at 19,200 bauds. Hence, the energy consumption behavior for this rate was less affected by factors such as the load effect given at low battery voltages or the delay in the CO${}_{2}$ sensor activation.

**Figure 5:**
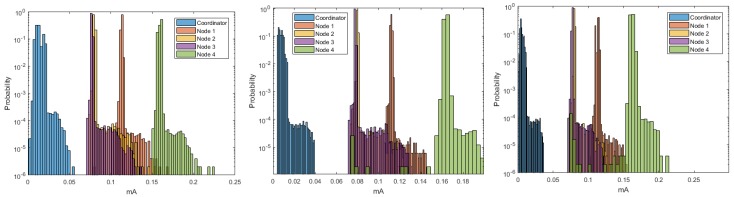
Histograms for DigiMesh network. From left to right: at 9600, 19,200, and 57,600 bauds.

Figure 5 shows the estimation of the histograms for the DigiMesh network at 9600, 19,200, and 57,600-baud rates. The elements of the DigiMesh network for the three speeds have the same current ranges with higher occurrence probability. In addition, the current range with lower probability of the Coordinator Node decreases with increasing speed. Accordingly, we identify that the sensor nodes have higher average energy consumption than the Coordinator Nodes, especially Sensor Node 4.

**Figure 6:**
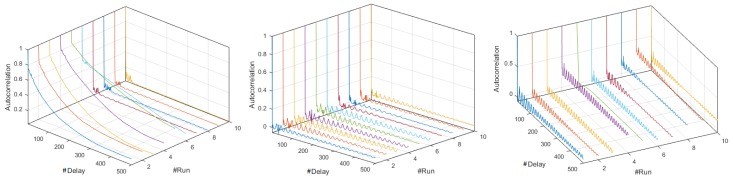
SAFs with different characteristics for DigiMesh network at 19,200 bauds. From left to right: Coordinator Node, Node 2, and Node 4.

Figure 6 shows relevant aspects identified in the autocorrelation analysis using the M-mode and the CI at 19,200- and 57,600-baud rates. The plots of the Coordinator Node at 57,600 and 9600 bauds were similar, and corresponded to a non-seasonal time process, since the decrease of their SAFs was slow and exponential-like. In contrast, the first six realizations of this node at 19,200 bauds recorded slow and anti-persistent drops, and for increasing run number, the battery voltage was reduced and the consumption signals tend to be uncorrelated processes, because the falls were rapid and with slight persistence in the initial delays. The persistence and fast descend of SAFs in Nodes 1 and 3 were similar for the three speeds, hence, we conclude that the consumption signals in these nodes is seasonal, independently of the transmission rate. The autocorrelation variations in the signals of Nodes 2 and 4 were rapid in all the runs and the number of signals with relevance throughout the delay was greater than 19,200 bauds, but in general terms we can classify the consumption of these nodes as uncorrelated processes.

**Figure 7:**
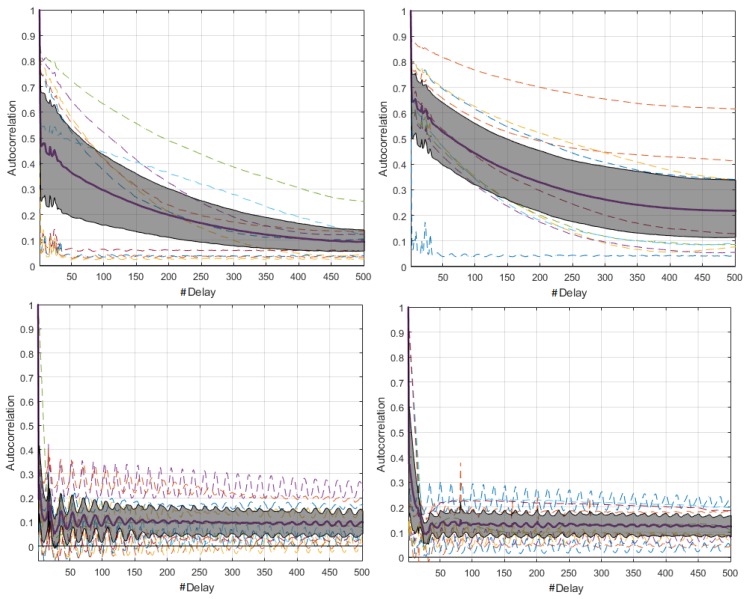
CI for SAF with distinctive characteristics for DigiMesh network: (**Left**) at 19,200 bauds; (**Right**) at 57,600 bauds; (**Top**) Coordinator Node; and (**Bottom**) Sensor Node 4.

Through the comparative study of the CI of the SAF for the three speeds, we identified relevant differences, which are shown in Figure 7. In the Coordinator Node, the consumption processes were on average seasonal for all speeds, as long as the battery was fully charged in each realization. In all cases, the widths of the CIs were greater with respect to the Sensor Nodes, and similarly at 9600 and 57,600 bauds. However, at 19,200 bauds the width decreased with increasing delay. In addition, the average SAF was higher at 57,600 bauds. The rapid and persistent drops, as well as the CI dimensions in the graphs of Nodes 1 and 3, were similar at all transmission rates, meaning that the consumption signals of these network elements are seasonal. The SAFs of Node 2 were uncorrelated at all speeds, as far as their values showed rapid drops without persistence with narrow CIs. Finally, the consumption signals from Node 4 were classified as non-correlated processes for all the tests. However, the speed increase caused a slight extension of the SAF in the first delays and the falls tended to be smoother. In addition, persistence was lost throughout the range of delays for the highest rate.

### 4.3. Energy Consumption Dynamics of ZigBee Networks

**Figure 8:**
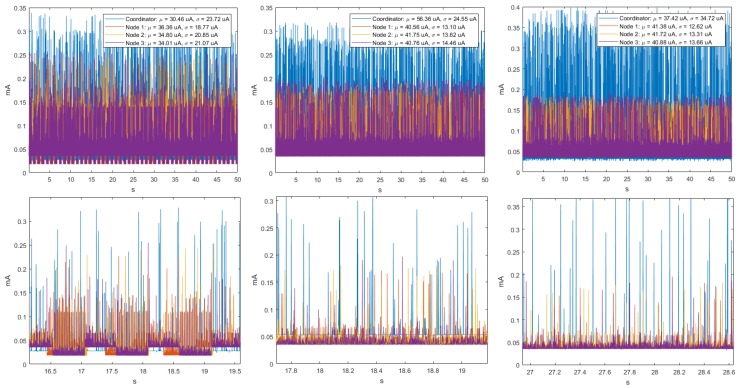
Time signals for ZigBee network. From left to right: at 9600, 19,200, and 57,600 bauds. (**Top**) Complete time signal; and (**Bottom**) time detail of the same signal.

The analysis of the time series in the ZigBee network at 9600, 19,200 and 57,600 bauds showed that, for the ten realizations, consumption fluctuated around the mean, there was no defined trend, and the variability remained stable. This also indicates that the behavior of the signals is homoscedastic. The cyclo-stationarity of the total and partial temporal signals for the 9600-baud rate is shown in Figure 8, together with the time signals for 19,200 and 57,600 bauds, which did not exhibit any periodicity. The μ and σ values of the Sensor Nodes indicate that the average energy consumption was the highest. The Coordinator Node had lower consumption, because it has the XBEE ZB S2 PRO communication module incorporated, and it is a low-power consumption module.

**Figure 9:**
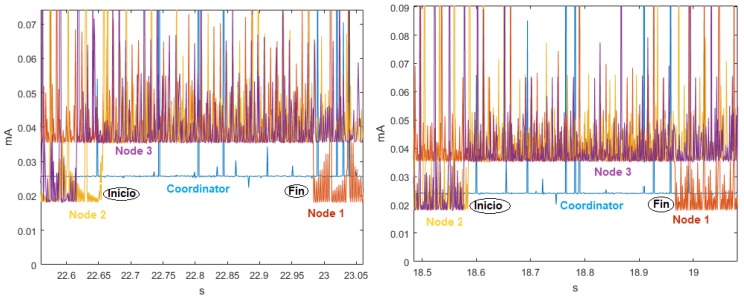
Complete time signals for ZigBee network at 9600 bauds in two example realizations: (**Left**) Realization 4; and (**Right**) Realization 7.

**Figure 10:**
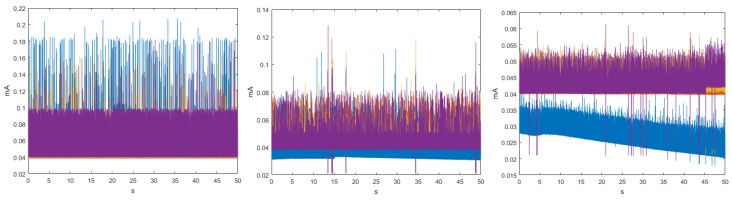
Time signals for ZigBee network at different NPLC. From left to right: 0.2, 2, and 10 NPLC.

Figure 9 shows at a closer scale the consumption peaks of two realizations for the speed of 9600 bauds, in which the signal periodicity can be identified. The peak pattern indicates that Node 2 initiates the communication, then Node 3, and later Node 1. The peaks of the Coordinator Node are present in the entire transmission sequence, whose duration is approximately 0.33 s. Five additional experimental tests were realized with the modification of the NPLC to characterize the periodicity for speeds of 19,200 and 57,600 bauds, however, it was not possible to visualize the presence of periodicity in any of them. The duration of the experiment was directly proportional to the resolution of the multimeter expressed in NPLC, for its highest value, and the acquisition of measurements lasted approximately 4 h per realization. In Figure 10, we observe that the increase of this parameter did not improve the perception of periodicity, on the contrary, the current in the Coordinator Node noticeably decreased. For this reason, we decided to maintain the NPLC to perform the energy consumption analysis for the ZigBee network.

Table 4 summarizes the average consumption and deviation of each realization for the three analyzed baud rates. The standard deviations were high, being above 12 μA at the Sensor Nodes and above 22 μA at the Coordinator Nodes. The transmission duration at 9600 bauds was to 0.33 s. This value was determined by taking the mode of the samples in each realization, and then calculating the average for all realizations.

**Figure 11:**
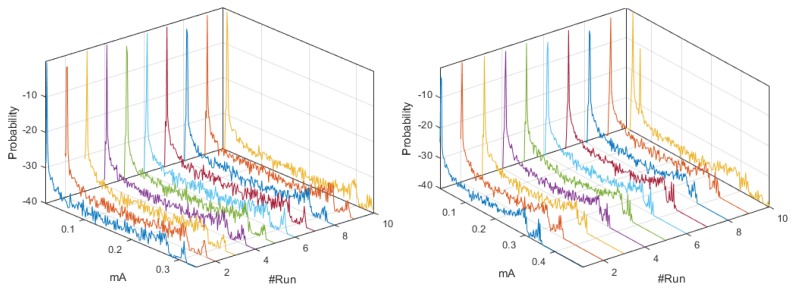
M-mode of *pdf* with distinctive characteristics for Coordinator Node of the ZigBee network: (**Left**) at 9600 bauds; and (**Right**) at 57,600 bauds.

**Figure 12:**
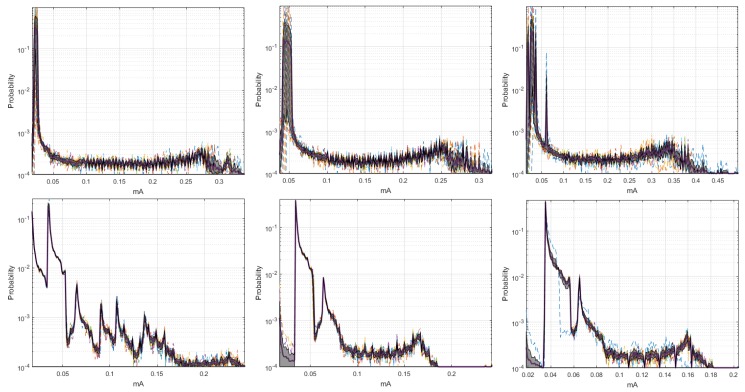

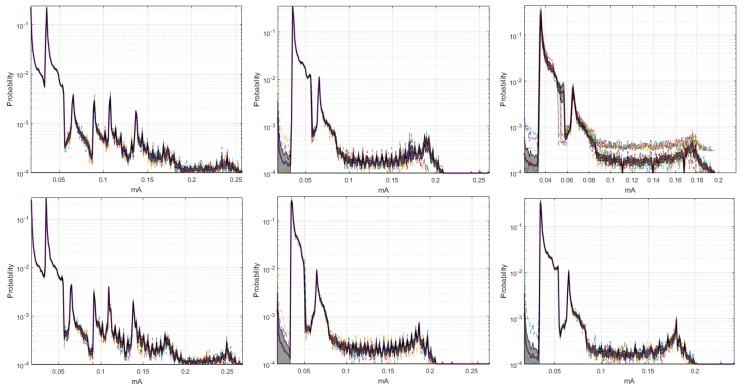
CI of *pdf* for ZigBee network. From left to right: at 9600, 19,200, and 57,600 bauds. From top to bottom: Coordinator Node and Nodes 1 to 3.

The similarity of the *pdf* signals obtained in ten realizations for the Coordinator Nodes was high between the speeds of 9600 and 19,200 bauds, but it was different by the slight displacement in Realization 10 at 57,600 bauds, as shown in Figure 11. In the case of the Sensor Nodes, the similarity was greater among the three baud rates. Then, we can say that the increase in the transmission speed does not significantly affect the behavior of the energy consumption. Figure 12 shows the estimated *pdf*, with their CI for the ZigBee network nodes, at the three speeds. The widest CIs were produced in the Coordinator Nodes, whereas the Sensor Nodes have narrow CI. The empirical *pdf* of the elements in the ZigBee network was multimodal, and it was skewed to the right. The *pdf* of the Coordinator Nodes were similar for the three speeds. The *pdf* of the Nodes 1, 2, and 3 at speeds of 19,200 and 57,600 bauds were similar, however, at 9600 bauds there were very low consumption values with very low probability that were apparently generated by electrical noise. In addition, we observed that the sensor nodes at low speed showed a greater number of modes, while the Coordinator Node at higher speeds had a greater number of modes than at low speeds. The range with the highest concentration of data consumption acquires an extension when increasing the transmission speed, which occurs only in the Coordinator Nodes.

**Figure 13:**
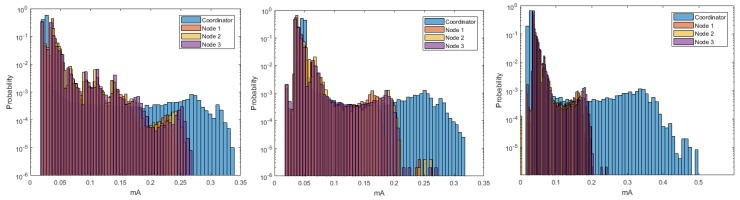
Histograms for ZigBee network. From left to right: at 9600, 19,200, and 57,600 bauds.

The estimated histograms of the ZigBee network for the three speeds are shown in Figure 13. Figure 13 (center, left) shows that the current ranges with higher probability of occurrence for the sensor and coordinator nodes were the same for the speeds of 9600 and 19,200 bauds. However, Figure 13 (right) exhibits a decrease in the current range with lower probability of occurrence for the sensor nodes, and this range increased for the Coordinator Node. Therefore, the speed increase to 57,600 implied a characteristic increase in the consumption peaks at the Coordinator Node. From these results, we conclude that the increase in the transmission speed to its maximum value increases, although not in a greater proportion, the average consumption of the node that manages the network.

**Figure 14:**
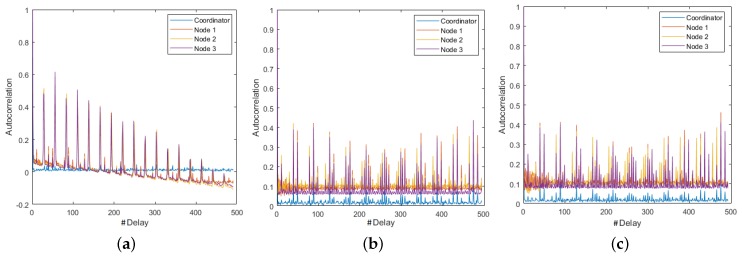

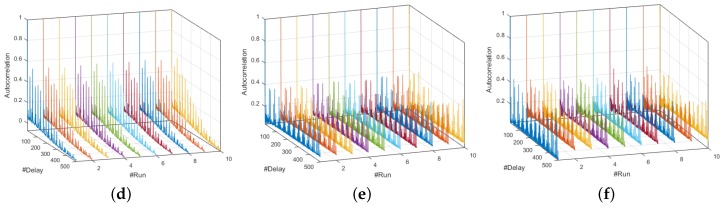
Representative SAFs for the ZigBee network. From left to right: 9600, 19,200, and 57,600 bauds. (**a**–**c**) Realizations 5, 1, and 8; and (**d**–**f**) M-modes of Sensor Node 1 at each speed.

From the review of the SAFs plotted in time series and with the M-mode for the three speeds, we conclude that for each node the resulting signals in each realizations were statistically similar. Figure 14 shows some representative SAFs. In Figure 14a–c, we observe that, for all members of the network, the fall was rapid and persistent. In the Coordinator Node, the persistence peaks were lower, and the correlation was nearby zero, which means that the relationship among observations was very low even in very short delays, and the results were quite similar for all speeds. Hence, the behavior of the SAFs in this node was independent of the transmission rate. In the sensor nodes, we note that for 9600 baud the correlation decreases with an approximately linear trend, and for delays larger than 180 ms, its values were negative. This is to say that the relation between a signal and its displacement is inverse. At 19,200 and 57,600 bauds, the SAF was positive and remains stable around the average. Figure 14d–f represents the M-mode plots of the SAF for Sensor Node 1 to corroborate that this different behavior between speeds was generated in the ten embodiments. The rest of the nodes were not shown because the SAF variations are very similar.

**Figure 15:**
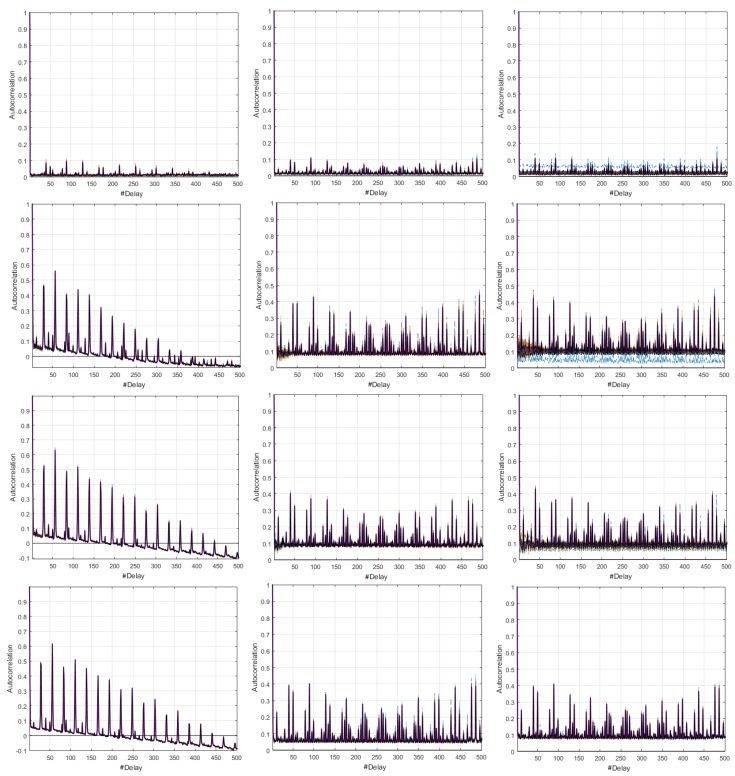
CI of SAF for ZigBee network. From left to right: 9600, 19,200, and 57,600 bauds. From top to bottom: Coordinator Nodes, and Nodes 1–3.

The characterization of energy consumption dynamics in terms of seasonality can be complemented by using the average SAF and its CI shown in Figure 15. For the Coordinator Node, the process can be described as uncorrelated for 9600 bauds, since the persistence of the function is lost with increasing delays. At high speeds it was stationary, since the SAF fall was fast and persistence was visible throughout the range of delays. The processes of Nodes 1–3 for 9600 bauds can be described as stationary for the first 200 delays, since the SAF falls are fast and the persistence is pronounced. After that, they were uncorrelated, and they invert due to the persistence, so that the correlation turns negative. In contrast, for 19,200 and 57,600 bauds, the processes were defined as stationary, regardless of the number of delays. Based on the small width of the CIs in all cases, we conclude that the average SAF of each node shows the presence or absence of seasonality in the signals with low accuracy. At the highest transmission rate, we observe a slight increase in the CIs for all the network elements.Finally, considering the narrow CI of the coordinator node, we conclude that the load effect does not affect the consumption signals, as happened in the DigiMesh network. In general terms, all the nodes in the ZigBee network at medium and high speeds would be the most suitable for energy consumption characterization over time.

### 4.4. Energy Consumption Dynamics of WiFi Network

**Figure 16:**
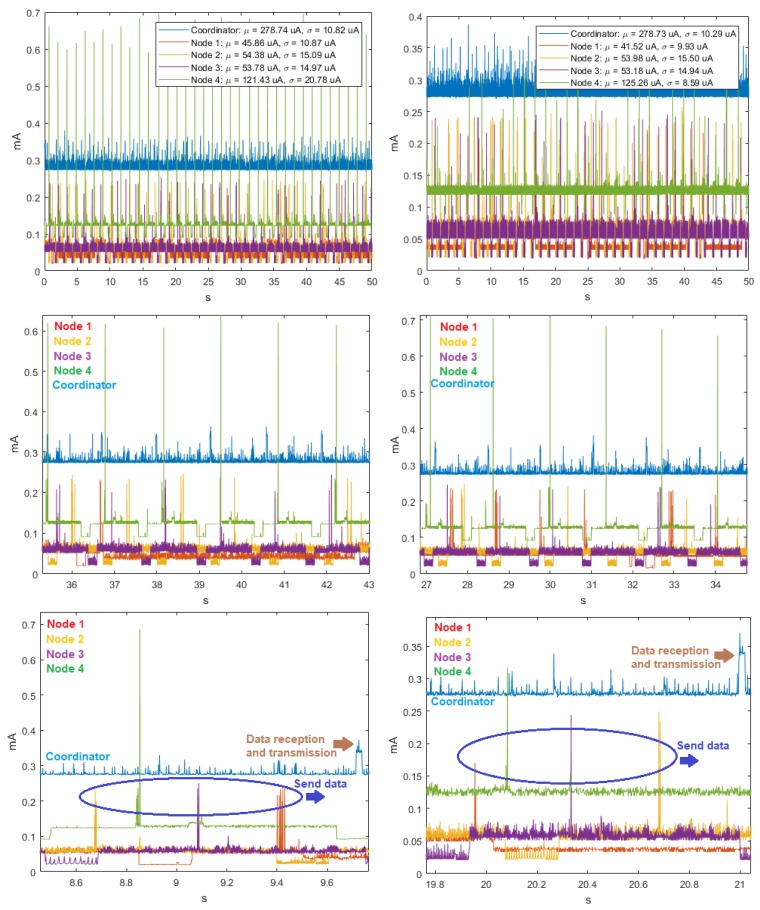
Time signals for WiFi network: (**Left**) at 9600 bauds; and (**Right**) at 57,600 bauds. From top to bottom: total time signal, time detail of the signal, and signal sections with closer detail.

The analysis of the time series of the ten realizations in the WiFi network at 9600- and 57,600-baud rates showed that, for all cases, consumption continued oscillating around the mean, therefore, there was no trend and the variability was less stable. This allows us to state that the behavior of the signals was mostly homoscedastic. Figure 16 shows the periodicity of the total and partial time signals of the consumption, and the pattern peaks of each node. The values of μ and σ at Node 4 indicated that its average energy consumption was higher, due to it having the CO${}_{2}$ sensor. The Coordinator Node had incorporated the RN-XV wireless communication module and the Meshlium™ router, so that its consumption was higher, because it is a device similar to a router for home wireless networks, and power saving is not a mandatory requirement. The consumption peaks of the sensor nodes occurred when the data sending was generated, and in the Coordinator Node at the moment of data reception and transmission. The event sequence occurred approximately every 1.2 s.

Table 5 shows the average consumption, as well as its deviation and the transmission duration values of each realization for two speeds. The average power consumption in Sensor Node 4 was the highest and in Node 1 it was the lowest. The average energy consumption of the Coordinator Node was maintained independently of the transmission rate. The standard deviation averages were high, about 20 μA at sensor nodes and about 11 μA at coordinator nodes. The transmission duration for both speeds was 1.28 s.

**Figure 17:**
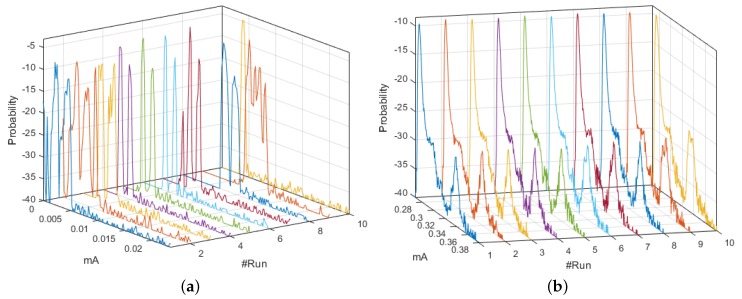
M-mode plots of the *pdf* with particular characteristics: (**a**) DigiMesh Coordinator Node at 57,600 bauds; and (**b**) WiFi Coordinator Node at 57,600 bauds.

The differences in the *pdf* between speeds were not significant. However, the results of the Coordinator Node (Meshlium™ WiFi router) differed considerably with respect to the DigiMesh network, as shown in the example in Figure 17. The signals of WiFi network were very similar in the ten realizations, since this device incorporates its own power system. In comparison to the DigiMesh network, the average consumption was not shifted between realizations, which means that this node was not affected by the load effect.

**Figure 18:**
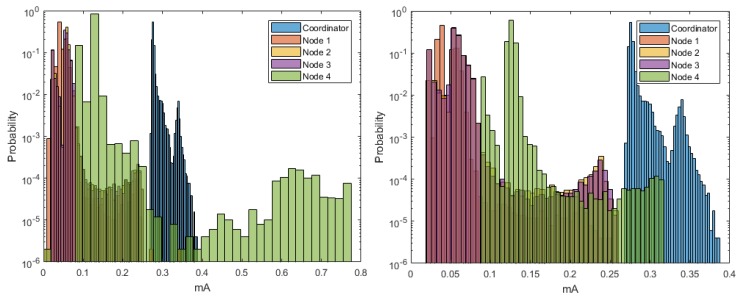
Histograms for WiFi network: (**Left**) at 9600 bauds; and (**Right**) at 57,600 bauds.

The estimation of the histograms represented in logarithmic scale for the 9600- and 57,600-baud rate is shown in Figure 18. For Nodes 1–3, and Coordinator, we observe that the current ranges with higher probability of occurrence were the same for both speeds. However, in Node 4, this range increased for the lower speed, which reveals the presence of current peaks with amplitudes slightly higher than those generated for 57,600 bauds. This behavior occurred for both speeds, and it was due to the type of sensor installed in this node, but it was more visible at 9600 bauds. In general terms, we conclude that these peaks do not significantly affect the average energy consumption, since their occurrence probability is low.

**Figure 19:**
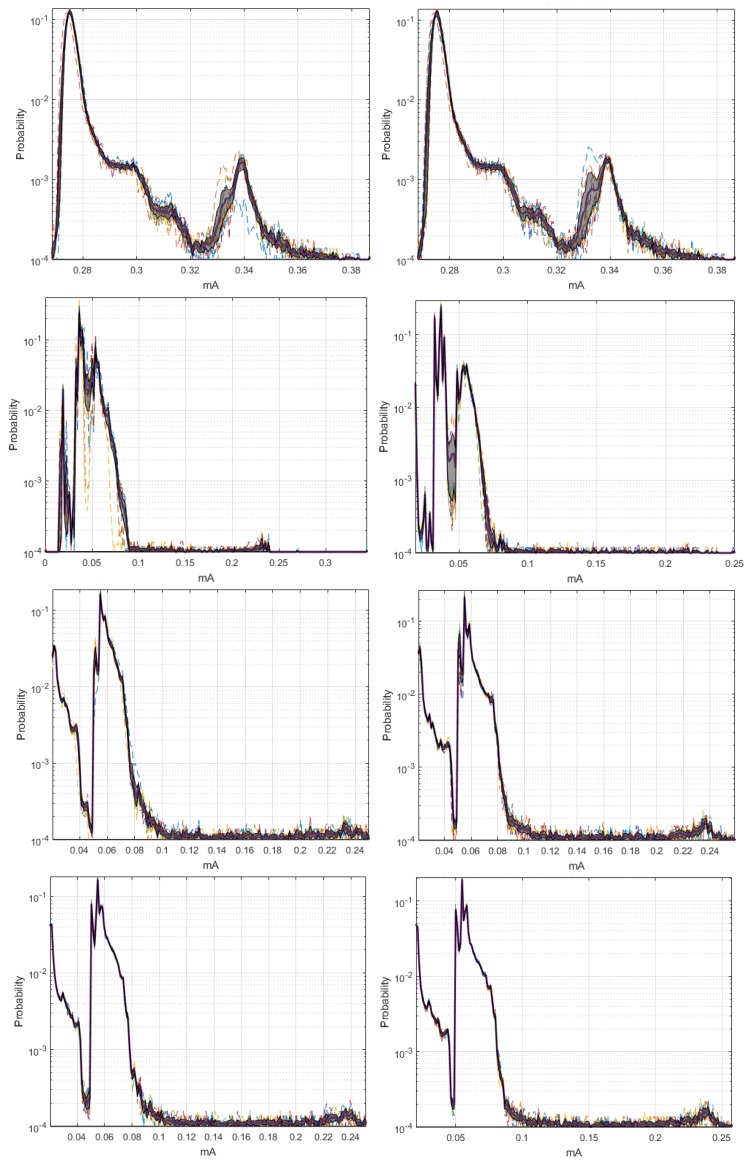

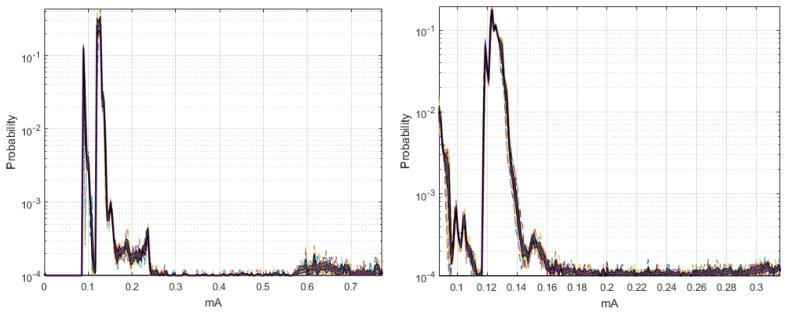
CIs of *pdf* for WiFi network: (**Left**) 9600 bauds; and (**Right**) 57,600 bauds. From top to bottom: Coordinator Node and Nodes 1–4.

Figure 19 shows the estimated *pdf*, with their respective CI for the WiFi network nodes, at 9600- and 57,600-baud rates. In all cases, the CIs were very narrow, and we conclude that the behavior of the energy consumption process in each node tends to be similar, regardless of the number of repetitions of the data collection experiment. The *pdf* in the Coordinator Node and in Nodes 1–3 were very similar for both speeds, since they maintained the multi-modal characteristic and the trend to skew to the right. In Node 4, we observe differences for consumption values lower than 0.1 mA, presumably generated by electrical noise. This was more perceptible at 9600-baud rate because the occurrence probability was high there. Besides, the electric current range covered by each *pdf* was reduced by increasing the speed. The *pdf* at 9600 bauds showed consumption values higher than 0.6 mA, whose occurrence probability was low, and it was likely caused by random electric current spikes that are typical in the CO${}_{2}$ sensor operation. Nevertheless, this particularity does not significantly affect the consumption.

After a comparative study of the *pdf* and CIs of the three networks, we conclude that the WiFi network exhibits relevant differences. In the Coordinator Node, the CIs were considerably narrow, the modes of the curve were easily distinguished, they do not vary sharply, and the range of the *pdf* consumption values contained was high. In Node 1, the CI widths and the probability curves were similar, but the consumption values tend to be low. The *pdf* in Nodes 2 and 3 differ with respect to the other two networks, because an additional mode was generated for consumption values lower than 0.04 mA, whose probability amplitude was considerable. This was likely produced by noise, and also the electrical current range with major occurrence probability was high. With respect to Node 4 of the DigiMesh network, the *pdf* and the CIs at 19,200 and 57,600 bauds were very similar in form, but the average consumption was high, while at 9600 bauds the differences are noticeable in terms of the CI width and the number of modes.

**Figure 20:**
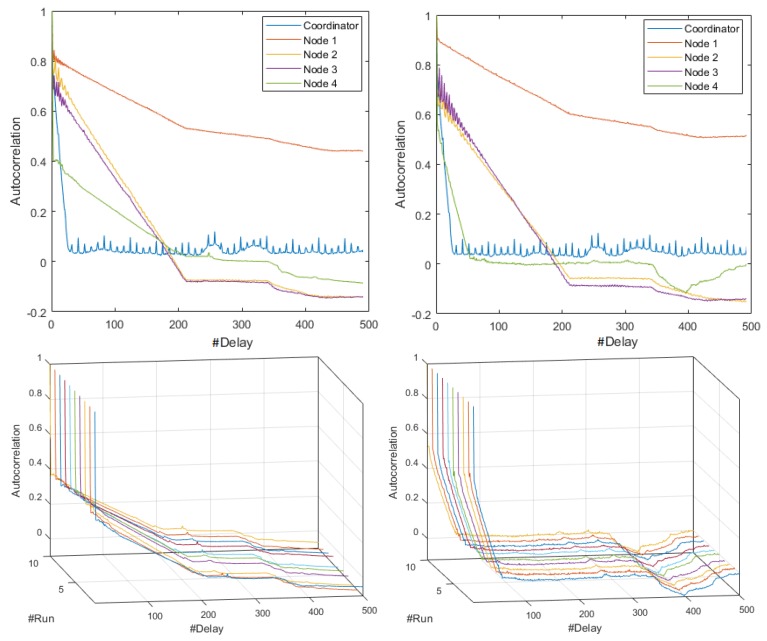
Representative SAF for WiFi network: (**Left**) 9600 bauds; (**Right**) 57,600 bauds; (**Top**) time series for Realizations 6 and 7; and (**Bottom**) M-mode of Sensor Node 4.

The estimation of the SAFs for the ten realizations revealed that the behavior of the functions for the Coordinator Node and for Nodes 1–3 is the same for the two speeds. Figure 20 shows some representative SAFs. The correlation of Node 1 falls slowly, with a slight persistence for the first 50 delays, its range was similar in all realizations and it was the highest with respect to the other nodes of this network. The SAFs in Sensor Nodes 2 and 3 were the same for both transmission rates, the fall was slow, and the persistence can be observed for delays less than 100. The correlation then decreases and reaches negative values near to zero with increasing delays. In this scenario, the relation between one observation and another was minimal and inverse. In the Coordinator Node, the SAF falls rapidly, the correlation was positive and it varies with persistence for all delays. However, its values were very low, therefore the relation between observations was very low even in the initial delays. In the case of Node 4, the SAF lacks persistence for both speeds, but the drop was less pronounced at 57,600 bauds and the increase in delays causes also a negative correlation. However, at the highest speed, we can see a slight increase for delays higher than 400, and if the delay continues to increase, the relation between observations tends to be high and direct.

**Figure 21:**
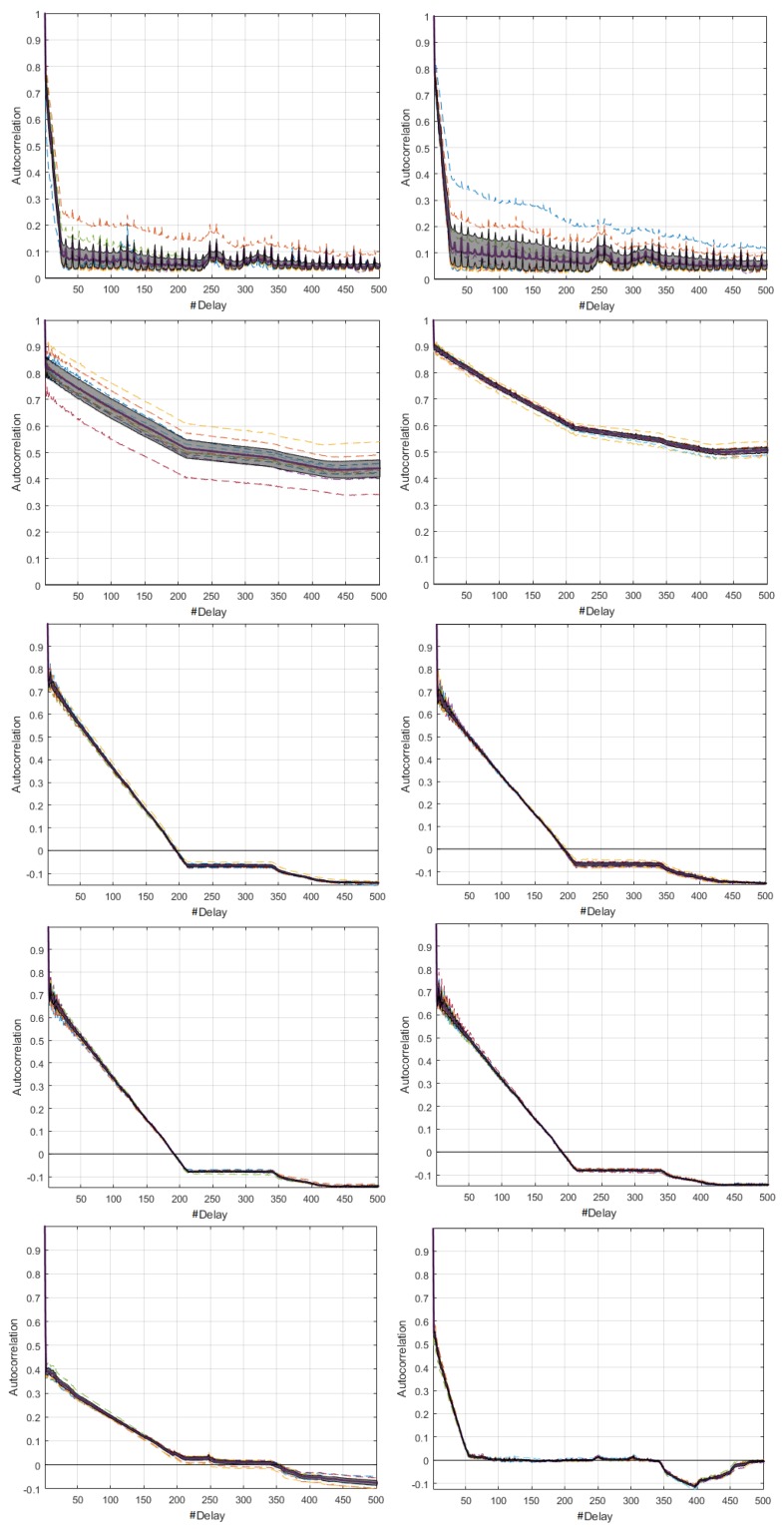
CI of SAF for WiFi network: (**Left**) 9600 bauds; and (**Right**) 57,600 bauds. From top to bottom: Coordinator, and Nodes 1–4.

By studying the average SAF and the CIs shown in Figure 21, and for both speeds, we complement the analysis of the energy consumption behavior of the elements of the network. The Coordinator Node was a seasonal process, given the rapid and persistent fall of SAF. For all sensor nodes, the processes were defined as non-seasonal, since the fall of the correlation coefficient was slow and it lacks persistence. Regarding the CIs, we conclude that, for Nodes 2–4, the widths were very small, and similar for the two transmission rates, while, in other network elements, we observe variations. For the highest speed, the CI amplitude increases in the Coordinator Node, and decreases in Node 1, but not in large magnitude, since the deviation does not exceed the 0.05. For Node 4, the fall of autocorrelation coefficient was more rapid, as well as its increase to delays greater than 400, particularities that were already explained. In general terms, the SAF average of each node defines with enough precision the presence or absence of seasonality in the signals, since the intervals were quite narrow.

## 5. Discussion and Conclusions

In this paper, we addressed the detailed study of the dynamics of energy consumption time processes in three types of WSNs, namely, ZigBee with star topology, ZigBee with mesh topology (DigiMesh), and WiFi with access point topology, at different transmission rates, for the monitoring of tomato greenhouses in Ecuador. The electrical current measurements of the nodes were acquired at a fast sampling rate, using high resolution multimeters that were connected to the data storage station through a local area network. The experiments were conducted with all the network elements being active, distributed in line of sight, and at the same distance. For the three networks, the test was repeated several times for each transmission rate, and without charging the battery between runs to analyze the impact of battery discharge over the reliability of the acquired consumption data. The consumption dynamics was scrutinized by using stochastic process analysis techniques, such as time series, *pdf*, histograms, and autocorrelation estimation, complemented with bootstrap resampling and CIs, and the results of all the realizations were simultaneously represented by the M-mode.

The power consumption of the WSNs nodes was evaluated considering the type of sensors, the network topology, the transmission speed, and the data transmission duration. According to the type of sensor, we conclude that the nodes with the CO${}_{2}$, wind speed, and wind direction sensors were the ones with the highest consumption, independently of the network communication technology. Regarding the network topology, we verify that the consumption is associated with the role of each node. The consumption of the sensor nodes was lower in the ZigBee and WiFi networks (star topology), where each node only communicates with its coordinator, in contrast to the DigiMesh network (mesh topology), where the links are redundant and the nodes communicate simultaneously with more than one network element. In the case of the coordinator nodes, we concluded that the consumption values were lower for the DigiMesh network, closely followed by the ZigBee network. The consumption of the WiFi coordinator node (Meshlium™ wireless router) was considerably greater than other networks, because the design of this device does not prioritize the issue of energy optimization. Based on these results, we conclude that, in terms of energy saving, the ZigBee network, with star topology and maximum transmission rate, was the most efficient option, and therefore the recommended one for greenhouse monitoring. The WiFi network can be considered as an appropriate second option, since the Coordinator Node is a device whose supply system is the commercial electrical network, and not a battery. Therefore, its high consumption is not a limitation, and the average consumption of the sensor nodes was very close to the measured ones in the ZigBee network. The DigiMesh network would be recommendable for applications where the redundancy of communication links is prioritized instead of energy saving.

A further objective of the present research was the characterization of energy consumption of WSNs nodes in terms of seasonality to identify the feasibility of its behavior prediction over time. Unlike the previous analysis, the seasonality was not significantly dependent on the type of sensors used in each node or the network topology, as the outcomes differed between networks, even for cases where sensor nodes measured the same variables. The results of the DigiMesh network were quite similar for the three speeds, i.e., the energy consumption processes were seasonal for the sensor nodes that measure solar and ultraviolet radiation, non-seasonal for the coordinating node, and weakly correlated for the CO${}_{2}$ sensor node. For the WiFi network at the different transmission rates, the consumption process of the coordinator node was seasonal, while for all the sensor nodes it was non-seasonal. The power consumption processes of all ZigBee network nodes were seasonal for the medium and high speeds, while for the minimum speed the seasonality was lost from approximately 200 milliseconds of delay. Based on this background, we conclude that the ZigBee network at medium and high transmission rates was the most recommendable for purposes of energy consumption modeling of greenhouse monitoring networks.

The relevant contributions of the energy consumption study developed for the sensor and coordinator nodes of three WSNs with different communication technologies, topologies, and transmission rate are described next:

- The usual patterns and duration times of each transmission were determined by locating the periodic spikes in the time series of the electrical current measurements.
- A detailed statistical description of the consumption dynamics and ergodic properties using histograms, the estimated *pdf*, and their CIs was elucidated.
- The nodes where consumption prediction is feasible by means of seasonality study (estimated SAF and CIs), as well as the differentiation of the nodes affected by the type of topology or speed change, were identified. To consider additional aspects such as the full charge of the battery during the experimental phase, a particular case of this study was given by the sensor nodes that measure CO${}_{2}$, as well as the Coordinating Nodes of the ZigBee and DigiMesh networks.
- Recommendation of the most suitable network in terms of energy saving for use in greenhouse monitoring systems are given.

In summary, this work provides engineers and professionals in the field with a set of guidelines that can be taken into account for the design and deployment of greenhouse monitoring networks, to choose the most appropriate technology and topology, as well as the ideal transmission rate in terms of energy consumption, without affecting the quality of the monitored data.

This research can be further expanded through the application of statistical learning and machine learning techniques to predict the trend of battery discharge, depending on wireless technology, network topology, transmission rate, link distances, and types of installed nodes.

## Figures and Tables

**Table 1:** State of the art of relevant works in WSN literature related with energy aspects.

Work	Technical Contribution
Barboni et al. (2008) [30]	An electronic system to visualize node current consumption usage, and charge extracted from the battery during node operating states.
Niewiadomska et al. (2009) [31]	A short overview of the energy conservation techniques and algorithms for calculating energy-efficient topologies for WSNs.
Al et al. (2010) [32]	A technique for WSN with mobile sensor nodes reducting 50% in the energy consumption.
Casilari et al. (2010) [33]	A full experimental characterization of current consumption in ZigBee sensor nodes.
Ishmanov et al. (2011) [34]	A review of energy consumption balancing (ECB) issues in WSNs.
Lozneanu et al. (2011) [35]	Energy mathematica model for each part of the wireless sensor node, which is adaptable to any sensor node.
Mihajlov et al. (2011) [36]	A performance evaluation of a WSN.
Soua et al. (2011) [37]	A review of different techniques to reduce the consumption of the sensor nodes.
Kaur et al. (2012) [38]	An overview of WSNs and a scenario based comparison for energy efficiency between different topologies.
Silva et al. (2012) [39]	A methodology to evaluate the dependability of WSNs in typical industrial environments.
Distefano (2013) [40]	An evaluation of the reliability of WSN applied from a system reliability point of view.
Deshpande et al. (2014) [41]	A study of the topology control to minimize energy consumption for a WSN.
Luo et al. (2014) [42]	Analysis of the distributions of the energy consumption for the communications among nodes.
Moschitta et al. (2014) [43]	A review on energy consumption measurements in WSN networks, highlighting the node architecture and the network operation.
Rault et al. (2014) [44]	A new taxonomy of energy conservation schemes and an analysis of how these techniques can affect the performance of their applications.
Abo et al. (2015) [45]	An energy consumption model for a WSN node based on physical and MAC layer parameters.
Aguirre et al. (2015) [46]	A radio planning analysis for WSN deployment is proposed by employing a deterministic 3D ray.
Zhu et al. (2016) [47]	A reliability evaluation model for network transmission.
Dâmaso et al. (2017) [48]	An integrated analysis of power consumption and reliability.

**Table 2:** Relevant parameters in the data collection of energy consumption.

Network	Nodes	NPLC	Noise RMS (PPM)	Baud Rate	Waspmote Card	Communication Module
ZigBee	1 Coordinator 3 Sensor Nodes	0.006	6	9600 19,200 57,600	Software: ID PRO Libelium™ Hardware: Not required	Software: X-CTU Hardware: ZigBee Gateway
DigiMesh	1 Coordinator 4 Sensor Nodes	2	0.2	9600 19,200 57,600	Software: ID PRO Libelium™ Hardware: Not required	Software: X-CTU Hardware: ZigBee Gateway
WiFi	1 Coordinator 4 Sensor Node	1	0.3	9600 57,600	Software: ID PRO Libelium™ Hardware: Not required	Software: FTDI drivers Microchip™ Hardware: RN-XV-EK1 Module

**Table 3:** Values of energy consumption and transmission duration for the DigiMesh network.

Baud Rate	Network Element	Parameters	1	2	3	4	5	6	7	8	9	10	Average
9600	Coordinator	μ (uA)	20.48	19.19	14.65	14.32	13.18	12.01	11.17	10.34	9.63	7.90	13.29
		σ (uA)	1.05	0.94	1.83	0.74	0.68	1.15	0.97	1.05	1.09	1.00	1.05
	Node 1	μ (uA)	112.11	112.34	112.54	113.03	113.14	113.44	113.58	113.99	114.29	114.14	113.26
		σ (uA)	0.43	0.50	0.41	0.45	0.53	0.64	0.59	0.43	0.42	0.46	0.49
	Node 2	μ (uA)	80.28	80.13	80.16	80.24	80.18	80.53	80.37	80.44	80.44	80.38	80.32
		σ (uA)	0.57	0.43	0.48	0.46	0.58	0.78	1.00	0.96	0.98	0.95	0.72
	Node 3	μ (uA)	77.01	77.17	77.23	77.25	77.2	77.18	77.37	77.37	77.3	77.26	77.23
		σ (uA)	0.71	0.60	0.71	0.78	0.63	0.70	0.59	0.59	0.71	0.58	0.66
	Node 4	μ (uA)	156.77	157.31	157.94	159.18	159.66	161.78	161.56	161.41	161.62	161.96	159.92
		σ (uA)	0.39	0.43	0.41	0.44	0.43	0.87	0.58	0.39	0.40	0.38	0.47
	Transmission Duration	T (s)	0.71	0.68	0.68	0.68	0.68	0.70	0.68	0.68	0.67	0.67	0.68
19,200	Coordinator	μ (uA)	10.78	9.55	8.61	7.61	7.40	7.57	6.02	5.42	4.87	4.40	7.22
		σ (uA)	1.30	1.33	1.31	1.35	1.49	1.07	0.69	0.71	0.67	0.74	1.07
	Node 1	μ (uA)	111.19	111.33	111.53	111.9	112.02	112.61	112.01	112.27	112.52	112.76	112.01
		σ (uA)	0.39	0.50	0.41	0.43	0.39	0.52	0.43	0.41	0.44	0.37	0.43
	Node 2	μ (uA)	79.98	79.98	79.97	80.04	80.04	80.03	80.2	80.03	80.08	80.13	80.05
		σ (uA)	0.52	0.46	0.46	0.50	0.46	0.49	0.59	0.71	0.64	0.59	0.54
	Node 3	ć (uA)	77.35	77.39	77.37	77.43	77.36	77.38	77.37	77.39	77.45	77.5	77.40
		σ (uA)	0.83	0.96	0.81	0.83	0.65	0.85	0.62	0.55	0.63	0.64	0.74
	Node 4	μ (uA)	161.51	161.47	161.57	164.57	165.25	164.59	163.50	164.05	164.46	164.86	163.58
		σ (uA)	0.32	0.39	0.42	0.38	1.69	0.43	0.36	0.46	0.42	0.41	0.53
	Transmission Duration	T (s)	0.68	0.68	0.68	0.67	0.67	0.67	0.68	0.68	0.68	0.68	0.68
57,600	Coordinator	μ (uA)	4.03	9.55	8.32	5.63	4.74	4.28	3.87	4.47	4.02	3.26	5.22
		σ (uA)	0.69	1.19	1.12	1.00	1.03	1.00	1.02	1.39	1.69	1.42	1.16
	Node 1	μ (uA)	112.72	112.99	113.33	113.71	114.94	115.15	115.33	115.47	110.00	110.21	113.39
		σ (uA)	0.44	0.49	0.46	0.40	0.45	0.42	0.48	0.46	0.47	0.41	0.45
	Node 2	μ (uA)	80.08	80.22	80.28	80.29	80.35	80.36	80.33	80.31	80.60	80.45	80.33
		σ (uA)	0.44	0.68	0.66	0.51	0.50	0.38	0.58	0.45	0.65	0.70	0.56
	Node 3	μ (uA)	77.51	77.21	77.42	77.59	77.66	77.65	77.64	77.59	76.84	76.98	77.41
		σ (uA)	0.76	0.61	0.65	0.62	0.58	0.55	0.57	0.54	0.79	0.62	0.63
	Node 4	μ (uA)	164.97	159.83	159.97	161.03	164.01	164.21	163.64	163.66	159.77	160.43	162.15
		σ (uA)	0.43	0.41	0.47	0.57	2.18	2.12	2.73	0.59	0.55	0.43	1.05
	Transmission Duration	T (s)	0.68	0.70	0.68	0.67	0.66	0.66	0.66	0.68	0.69	0.68	0.68

**Table 4:** Values of energy consumption and transmission duration for the ZigBee network.

Baud Rate	Network Element	Parameters	1	2	3	4	5	6	7	8	9	10	Average
9600	Coordinator	μ(uA)	30.46	30.11	28.88	28.29	27.8	27.26	26.64	26.2	25.72	25.22	27.66
		σ (uA)	23.72	23.89	22.91	22.44	23.06	22.63	22.42	22.25	22.53	22.22	22.81
	Node 1	μ (uA)	36.36	36.29	36.31	36.11	36.03	35.95	35.94	35.85	35.93	35.85	36.06
		σ (uA)	18.77	18.54	18.71	18.63	18.38	18.72	18.59	18.3	18.54	18.51	18.57
	Node 2	μ (uA)	34.8	34.84	34.97	34.88	34.82	34.71	34.76	34.7	34.71	34.66	34.79
		σ (uA)	20.85	20.7	20.76	20.59	20.58	20.56	20.55	20.5	20.38	20.5	20.60
	Node 3	μ (uA)	34.01	34.08	34.31	34.32	34.31	34.08	34.11	33.98	34.03	34	34.12
		σ (uA)	21.07	21.17	21.35	21.35	21.15	21.3	20.95	20.95	21.02	21.09	21.14
	Transmission Duration	T (sg)	0.43	0.26	0.34	0.42	0.29	0.42	0.29	0.24	0.32	0.32	0.33
19,200	Coordinator	μ (uA)	56.36	54.83	53.59	52.54	51.5	50.43	49.44	48.31	47.3	46.36	51.07
		σ (uA)	24.55	23.12	22.62	22.58	22.97	22.56	22.44	22.32	22.92	23.01	22.91
	Node 1	μ(uA)	40.56	40.6	40.28	40.45	40.66	40.61	40.58	40.61	40.58	40.66	40.56
		σ (uA)	13.1	13.15	12.89	12.89	12.79	12.88	12.71	12.78	12.61	12.87	12.87
	Node 2	μ (uA)	41.75	41.76	41.63	41.94	41.94	41.88	41.93	41.92	41.9	41.86	41.85
		σ (uA)	13.62	13.89	15.47	15.65	14.86	14.92	14.86	14.86	14.83	14.75	14.77
	Node 3	μ (uA)	40.76	40.7	40.51	40.87	41.28	41.13	40.98	40.98	41.1	40.99	40.93
		σ (uA)	14.46	14.26	14.67	14.45	14.49	14.44	14.43	14.22	14.42	14.43	14.43
57,600	Coordinator	μ (uA)	44.63	37.42	36.57	35.53	34.26	33.28	32.17	30.97	24.75	24.28	33.39
		σ (uA)	37.98	34.72	34.1	35.54	33.94	33.92	33.64	33.02	30.87	31.14	33.89
	Node 1	μ (uA)	41.21	41.38	41.39	41.4	41.35	41.3	41.29	41.28	41.13	41.16	41.29
		σ (uA)	11.67	12.62	12.83	12.76	12.78	12.9	12.88	12.93	12.51	12.56	12.64
	Node 2	μ (uA)	41.57	41.72	41.85	41.84	41.37	41.27	41.24	41.27	41.86	41.85	41.58
		σ (uA)	13.71	13.31	13.93	13.01	13.44	13.96	13.58	13.4	13.65	13.63	13.56
	Node 3	μ (uA)	40.62	40.88	41.03	41.14	41.15	41.15	41.12	41.43	41.45	41.52	41.15
		σ (uA)	14.59	13.66	14.01	14.04	13.93	13.91	13.53	14.04	13.98	13.94	13.96

**Table 5:** Values of energy consumption and transmission duration for the WiFi network.

Baud Rate	Network Element	Parameters	1	2	3	4	5	6	7	8	9	10	Average
9600	Coordinator	μ (uA)	277.99	278.06	278.43	278.74	279.25	278.63	278.73	278.83	278.65	278.49	278.58
		σ (uA)	7.47	11.87	10.32	10.82	11.29	10.53	10.29	10.52	10.6	10.23	10.39
	Node 1	μ (uA)	45.03	43.39	44.82	45.86	46.15	45.57	46.55	45.24	44.28	42.46	44.94
		σ (uA)	10.34	10.28	10.71	10.87	11.18	10.74	10.89	10.87	11.05	10.2	10.71
	Node 2	μ (uA)	55.25	54.29	54.47	54.38	54.27	54.03	54.13	53.89	53.8	53.79	54.23
		σ (uA)	15.48	15.24	14.74	15.09	14.93	14.62	14.73	14.51	14.66	14.7	14.87
	Node 3	μ (uA)	53.35	276.18	53.43	53.78	53.58	53.44	53.46	53.3	53.35	53.48	75.74
		σ (uA)	15.28	10.42	15.04	14.97	14.9	15.13	15.13	15.43	15.37	15.16	14.68
	Node 4	μ (uA)	123.63	120.98	120.68	121.43	121.43	121.8	123.38	123	123.08	123.3	122.27
		σ (uA)	22.73	20.73	20.12	20.78	20.78	21.03	21.83	22.66	22.44	22.71	21.58
	Transmission Duration	T (sg)	1.29	1.28	1.28	1.28	1.27	1.30	1.30	1.27	1.28	1.29	1.28
57,600	Coordinator	μ (uA)	279.01	278.06	278.43	278.74	279.25	278.63	278.73	278.63	278.66	278.49	278.66
		σ (uA)	13.02	11.87	10.32	10.82	11.29	10.53	10.29	10.52	10.6	10.23	10.95
	Node 1	μ (uA)	41.99	42	42.41	42.18	41.5	41.28	41.52	42.07	42.15	42.46	41.96
		σ (uA)	10.22	10.22	10.42	10.37	9.74	9.71	9.93	10.12	10.22	10.2	10.12
	Node 2	μ (uA)	54.72	54.13	54.18	54.25	53.77	53.78	53.98	53.62	53.79	54.12	54.03
		σ (uA)	15.3	15.64	15.38	15.44	15.38	15.52	15.5	15.33	15.52	15.37	15.44
	Node 3	μ (uA)	53.48	53.78	53.44	53.34	53.42	53.35	53.18	53.07	53.34	53.11	53.35
		σ (uA)	15.36	15.66	15.63	15.37	15.65	15.64	14.94	15.23	15.26	15.15	15.39
	Node 4	μ (uA)	123.75	123.78	124.26	124.42	124.89	124.8	125.26	125.04	125.04	125.23	124.65
		σ (uA)	8.64	8.41	8.49	8.88	9.29	8.85	8.59	8.83	8.83	8.84	8.77
	Transmission Duration	T (sg)	1.27	1.28	1.28	1.28	1.28	1.3	1.29	1.29	1.27	1.29	1.28

**Figure A1:**
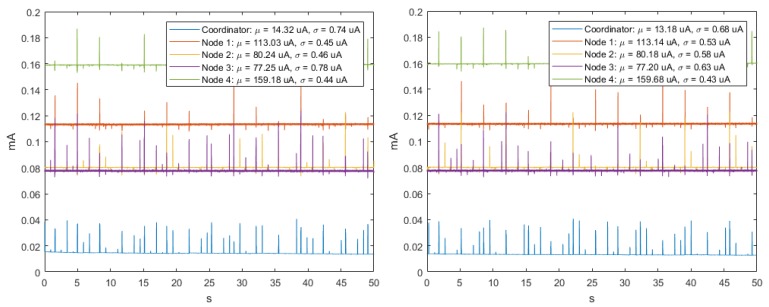
Detailed time signals for DigiMesh network at 9600 bauds in two example realizations: (**Left**) Realization 4; and (**Right**) Realization 5.

**Figure A2:**
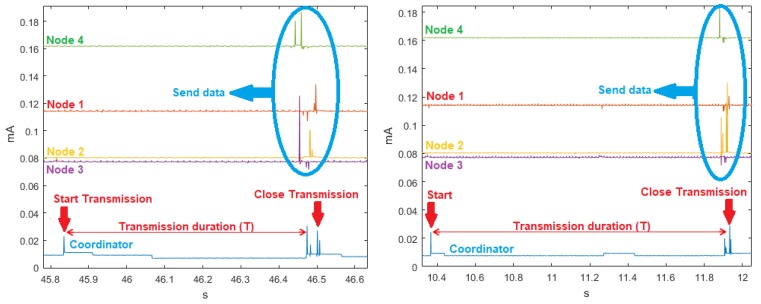
Closer details signal sections for DigiMesh network, and spike sequence of two realizations at 9600 bauds: (**Left**) Realization 9 with *T* = 0.68 s; and (**Right**) Realization 10 with *T* = 1.57 s.

**Figure A3:**
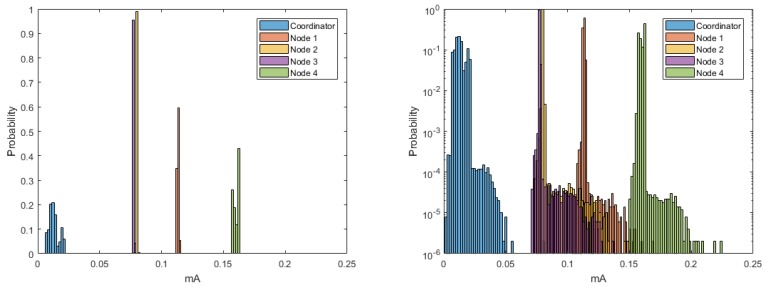
Histograms of the consumption signals for DigiMesh network at 9600 bauds: (**Left**) linear scale; and (**Right**) logarithmic scale.

**Figure A4:**
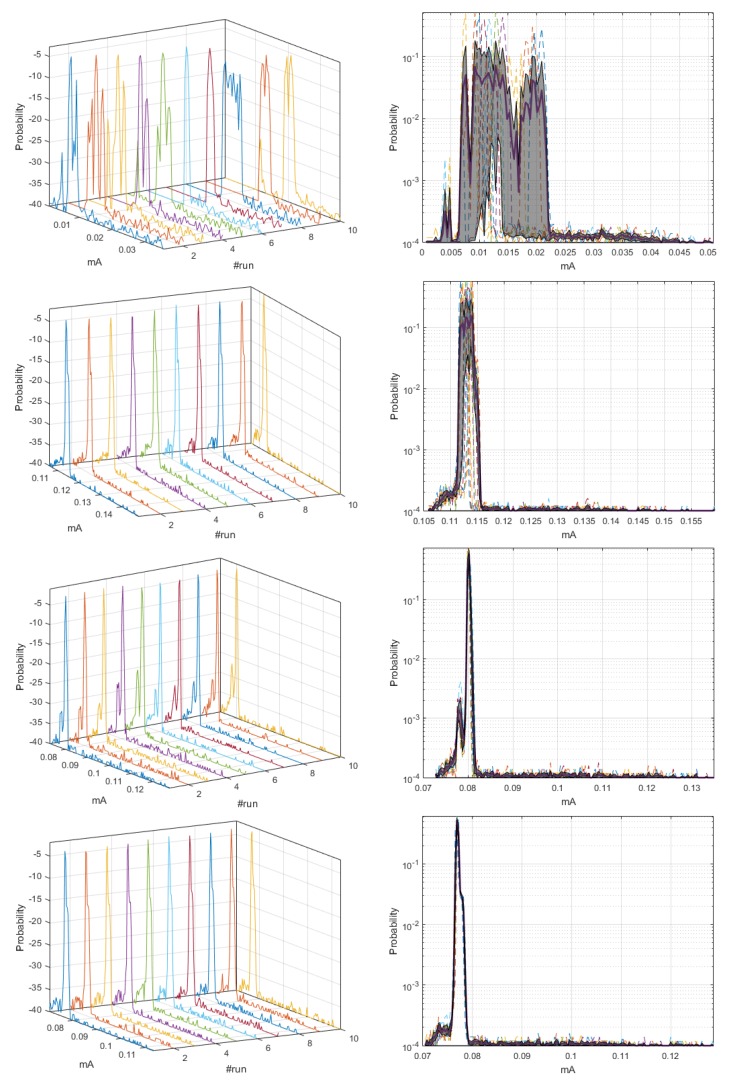

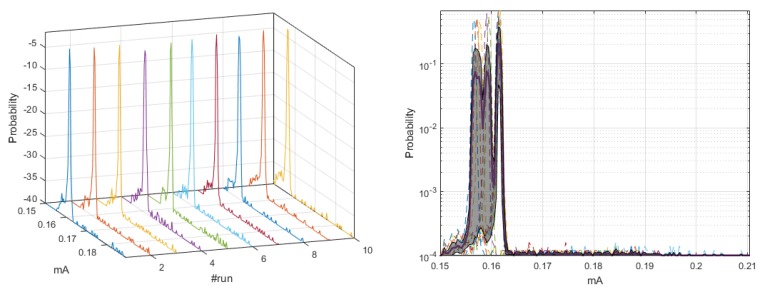
Plots of *pdf* for DigiMesh network at 9600 bauds: (**Left**) M-mode; and (**Right**) histograms of each realization (discontinuous), and bootstrap CI (gray shading). From top to bottom: Coordinator Node and Nodes 1–4.

**Figure A5:**
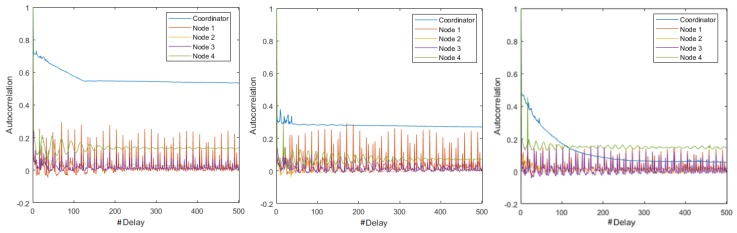
Time signals of SAF for DigiMesh network at 9600 bauds. From left to right: Realizations 1, 4, and 7.

**Figure A6:**
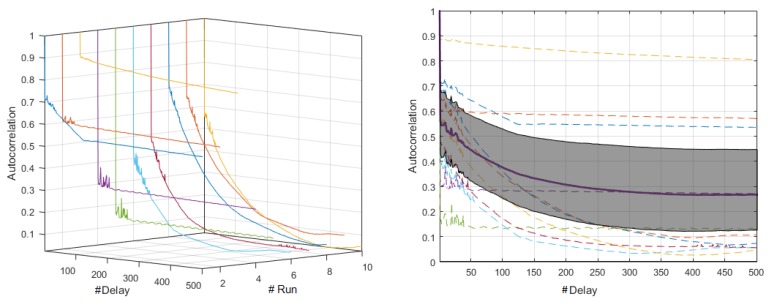

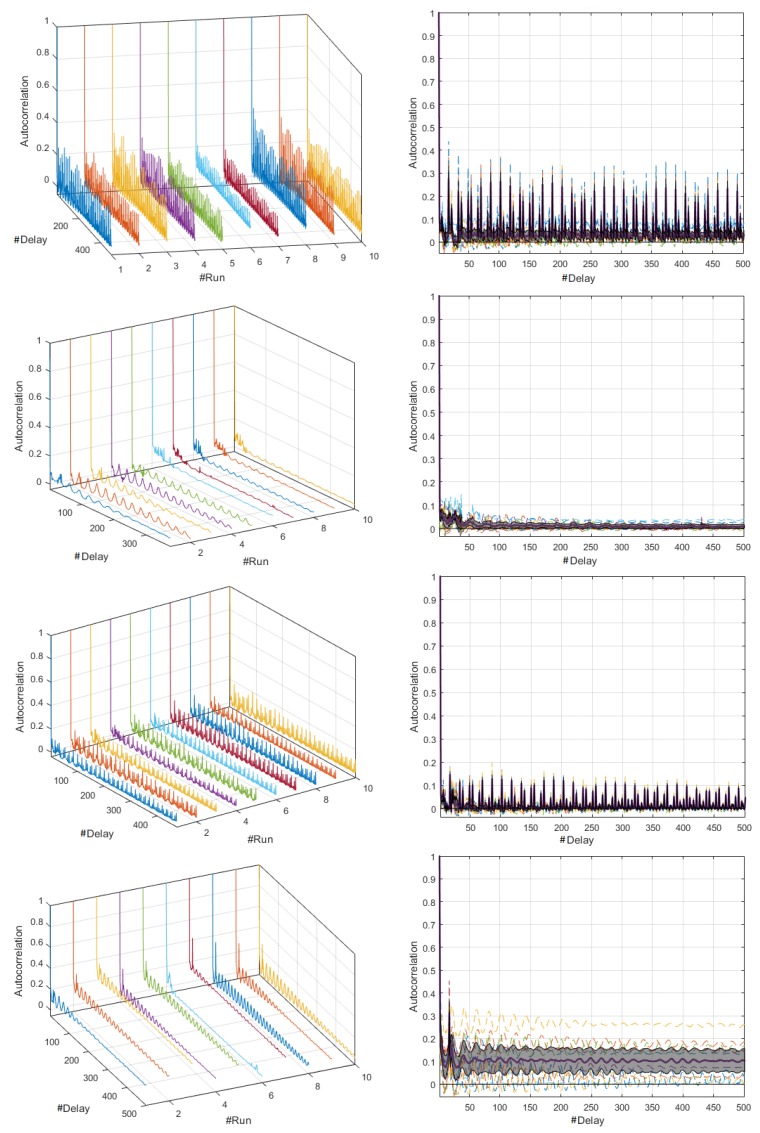
Plots of SAF for DigiMesh network at 9600 bauds: (**Left**) M-mode; and (**Right**) autocorrelograms of each realization, and the bootstrap CI. From top to bottom: Coordinator and Nodes 1–4.

## Appendix A

*Time Series Notation and M-Mode Plots for Different Runs.* The energy consumption signals in WSN nodes are temporal random processes, since they are defined as time functions at some observation interval, and it is not possible to describe exactly their waveform as it will be observed in the future before performing another experiment. The recorded temporal signals are defined as continuous stochastic processes, and we can denote each of these processes as φ(t) [57]. In general terms, the mathematical notation assigned to an acquired consumption signal is x(t), and each signal generated to $j^{th}$ realizations is defined as $x_{j}\left(t\right)$, with j=1,2,...,R, where *R* is the total number of realizations. All signals are observed in a time interval given by $\left(t_{i},t_{f}\right)$ with duration *T*, and each of these measured signals is denoted as

(A1) $$x_{j}\left(t\right)=\left\{\begin{array}{cc} x(t) & \mathrm{if}\;t\in (t_{i},t_{i}+T)\\ 0 & \mathrm{otherwise} \end{array}\right.$$

The temporal evolution of a stochastic process in a sample period $T_{s}$ is analyzed using discrete-time series, which are also called *sampled signals*. The sampling process transforms a band-limited continuous-time signal into a series of amplitude values at discrete-time instants [58]. Sampled signals can be obtained with the classical impulse- and uniform-sampling technique, which multiplies the input signal by a train of unit impulses in which the intervals between consecutive impulses are the equidistant sampling times, as described by

(A2) $$\Delta_{j}\left(t|T_{s}\right)=x_{j}\left(t\right)\cdot \sum_{n=-\infty }^{\infty }\delta \left(t-nT_{s}\right)$$

where the Dirac’s delta function or continuous-time unitary-impulse function is denoted as δ(t)[59]. Note that $x_{j}\left(t\right)$ is limited in duration, so that $\Delta_{j}\left(t|T_{s}\right)$ has a limited set of *N* possibly non-zero samples.

Based on the description of process φ(t) in the time domain, we can obtain its estimated average from the set of samples available for the *j* realizations by

(A3) $${\bar{\Delta }}_{j}\left(t|T_{s}\right)=E\left[\Delta_{j}\left(t|T_{s}\right)\right]$$

In addition, for a signal x(t), we can define an M-mode by defining a convenient second dimension *d*, so that Γ(t,d) denotes this representation in general terms. For instance, if the second dimension is the index corresponding to the $j^{th}$ realization, we can define the M-mode of the sampled process as a index-temporal and simultaneous representation of the realizations of the stochastic process as follows,

(A4) $$X\left(j,t\right)=x_{j}\left(t\right)$$

where the convention of capital notation is used for the M-mode of the represented signal.

*Probability Density Function and Bootstrap Averaging.* The study of the probability density function (*pdf*) of a continuous random process φ(t) is relevant because it quantifies the certainty and the uncertainty of the results obtained in each realization. The value of this function is positive throughout the domain [60]. Since $x_{j}\left(t\right)$ is a continuous random signal, we can define the pdf of its amplitude as $f_{x_{j}}\left(x\right)$, and it represents the overall distribution of its amplitudes, integrated with respect to time and then independently from it.

M-mode plots were also used in this work to represent jointly the estimated *pdf* of each realization, as denoted by:

(A5) $$F\left(j,f_{x_{j}}\right)=f_{x_{j}}\left(x\right)$$

where again the convention of capital is used for the M-mode of the *pdf*. If we want to obtain the overall estimation of process φ(t), denoted as $f_{x}\left(x\right)$, we can average in the available realizations, as denoted by

(A6) $$\bar{f}\left(x\right)=E_{j}\left[f_{x_{j}}\left(x\right)\right]$$

Bootstrap resampling can provide us with nonparametric estimation of a variety of CI for different estimations with non-trivial analytical estimation equations. By using bootstrap resampling, we generate b=1,...,B resamples of the empirical *pdf* by sampling with replacement of the process time-samples, which is denoted as $f_{x_{j}}^{*}\left(x,b\right)$. By following the usual bootstrap notation, the asterisk (∗) is usually applied to represent the bootstrap versions of the statistical elements obtained from the empirical process, so that $x_{j}{}^{*}\left(b\right)$ are set of the samples with replacement obtained from a given $j^{th}$ time series, and *B* is the total number of built resamples (often B=100, B=500, or B=10,000). Once resample *b* is created, a bootstrap replication can be obtained for any estimated statistical parameter, in our case the empirical *pdf*, in virtue of the plug-in principle, and with this we define its estimator as

(A7) $$\bar{f_{j}^{*}}\left(x,b\right)=E_{j}\left[f_{x}{}_{j}{}^{*}\left(x\right)\right]$$

The CI for the estimated *pdf* can now be easily obtained by using ordered statistics from the representation of the *B* replications. In our case, we used a significance level of 0.05.

*Simple Autocorrelation and its Bootstrap Average.* If $x_{j}\left(t\right)$ is the energy consumption signal of *j*th realization, then $x_{j}\left(t+\tau \right)$ represents its replica displaced by delay τ, and its SAF is calculated by

(A8) $$r_{x_{j}x_{j}}\left(\tau \right)=\int_{-\infty }^{\infty }x_{j}\left(t+\tau \right)x_{j}\left(t\right)dt$$

The M-mode of the autocorrelation functions of the *j*th realizations is then just given by

(A9) $$R_{x_{j}}\left(j,\tau \right)=r_{x_{j}x_{j}}\left(\tau \right)$$

The estimated average of the SAF for the *j*th realization is given by

(A10) $${\bar{r}}_{x_{j}x_{j}}\left(\tau \right)=E_{j}\left[r_{x_{j}x_{j}}\left(\tau \right)\right]$$

Similar to the analysis in the previous paragraphs, we can estimate the statistical distribution of the SAF and define its CI by using the bootstrap resampling method for nonparametric processes. The empirical SAF that we built for resample *b* is denoted by

(A11) $$r_{x_{j}x_{j}}^{*}\left(\tau ,b\right)=\int_{-\infty }^{\infty }x_{j}^{*}\left(t+\tau \right)x_{j}^{*}\left(t\right)dt$$

and using the *B* built resamples, we can calculate a bootstrap replication for the SAF distribution, by means of the plug-in principle, which is finally estimated as

(A12) $${\bar{r}}_{xx}^{*}\left(\tau ,b\right)=E_{j}\left[r_{x_{j}x_{j}}^{*}\left(\tau \right)\right]$$

The CI for the estimated SAF can again be obtained by using ordered statistics from the representation of its *B* replications, and again we used a significance level of 0.05.

## Appendix B

Here, we explain in detail the *methodology of energy consumption analysis* using as an example the DigiMesh network at a transmission rate of 9600 baud. This explanation extends to the analysis of the other case studies. Table A1 summarizes the applicability of the different statistical methods used in this study for the acquired electrical current signals.

**Table A1:** Summary of statistical tools used for consumption dynamics analysis.

Statistical Plot	Applicability
Time series	To determine trends, average, and variability of the energy consumption for each experiment run.
	To identify the approximate transmission patterns (in terms of consumption peaks repeated after some time intervals).
	To determine the duration time of each transmission (T).
Histograms and *pdf*	To represent the relative frequency distribution of consumption in the network nodes.
	To identify scattered data from the central distribution (tails with errors or noise).
	To identify multimodalities.
Estimated SAF	To determine persistence and stationarity profiles of the energy consumption processess.
CIs	To provide with confidence limits on histograms and SAFs.
M-mode	Simultaneous representation of the time processes, *pdf* and SAFs for each experiment.
	To better identify similarities and differences of the results accross runs.

The revision of the time series of the ten repetitions in the DigiMesh network at 9600-baud rate showed that, for all cases, consumption remains oscillating around the mean. Hence, there is no actual trend, and the variability remains stable, which allows us to say that the behavior of these signals was mostly homoscedastic. Figure A1 depicts two example observations, in which the value of the mean (μ) and the standard deviation (σ) of each node are also included. In addition, since the CO${}_{2}$ sensor of Node 4 requires greater current for its operation compared with the sensors installed in the other nodes, the average energy consumption of this node was notably higher. The coordinator incorporated only the communication module XBEE ZB S1 PRO, which is manufactured with ultra-low power technology, hence its consumption was lower.

The patterns of the spikes in each node were analyzed in detail in each series. In the absence of interaction among the elements of the network, their consumption remains almost constant around the average. However, when the communication is established, the consumption peaks are generated with different amplitudes and at time spacings, although they were very similar in terms of sequence of events. Figure A2 shows in an increased scale the consumption peaks of two realizations taken as an example at different time instants. Note that the Coordinator Node opens the communication channel and generates a data request to the four Sensor Nodes, then each node sends the information of the variables, and finally the Coordinator generates a closing communication message. The average transmission duration *T* obtained after visual analysis of all realizations varied approximately between 0.6 and 1.7 s.

The dynamics of consumption signals in the time domain was studied by using three of the methods described in Section 3. The M-mode proposed in this work provided us with simultaneous graphical representation of the empirical *pdf* for the energy consumption of the ten realizations performed for each node. This way, the similarity of the signals obtained between one and another realization was analyzed to validate the acquired data, discriminate the erroneous signals, and repeat the data collection process if necessary. The regions with the highest concentration of consumption measures for each node, as well as their variability and dispersion, were scrutinized with the histograms, so that an approximate view of the probability distributions was obtained. Finally, the graphs of the CI allowed analyzing the variability of the probability distributions, and, for this purpose, bootstrap techniques for each node yielded the CI for its estimated average, with 95% of confidence level. Each graph was studied in the linear and logarithmic scales, as shown in Figure A3, where the histograms represented with the second option can be visualized with better detail because transitions and tails are imperceptible in the linear coordinate axis. Then, it is more appropriate to analyze the histograms represented in logarithmic scale, since they expand the visual scope of the analysis.

Figure A4 depicts the M-mode signals for the empirical *pdf* of the ten realizations, and their CI obtained in each node. It can be seen that the similarity of the signals obtained in ten realizations for the Sensor Nodes was larger than for the Coordinator Node, since this was connected to the computer and an impedance mismatch was generated. This load effect was evidenced also in the time signals of the ten realizations, where the average of consumption decreased as the number of runs increased. Namely, if the battery voltage decreases, the similarity between the signals reduces, and for this reason it is recommended that the battery is charged to its maximum capacity in each realization.

The widest CI occurs in the Coordinator Node, followed by Node 4. Based on this, it was established that the behavior of the consumption of these nodes was not repetitive, and that it strongly depends on external factors. In the case of the Coordinator Node, the consumption was conditioned by the battery voltage and the severity of the mismatch with the load, whereas at Node 4 it is a function of the response time of the CO${}_{2}$ sensor. The CI of Nodes 1, 2, and 3 were narrower, which suggests that the consumption process in each transmission is similar and independent of the battery charge status. The empirical *pdf* of elements in the DigiMesh network was multimodal and biased to the left. The Coordinator Node is the network manager that interacts with all nodes. Based on this, and considering that the energy consumption of the transmission sequences is different for each sensor node, we verified that the distribution was quite spaced and with a greater number of modes than the rest of nodes. For sensor nodes, both the number and range of modes depend on the type and number of installed sensors.

The seasonality of consumption signals was analyzed using the SAF for 500 delays of one millisecond. By means of the representation of the SAF in time series, we can observe the correlation of all nodes, and for each realization. Figure A5 shows some signals on a linear scale that we selected as representative. In general terms, the SAF of the Coordinator Node falls slowly and exponentially, while, in the sensor nodes, the falls are fast, with persistence for Nodes 1, 3, and 4, and anti-persistence in Node 2. With respect to the average of the correlation coefficient, in the Coordinator Node and in Sensor Nodes 1, and 4, it varies significantly in some realization, because of the load effect already explained. In the rest of the nodes, the average was about the same in all the realizations and its value was nearby zero for larger delays, hence we deduce that the relation between a signal and its out-phase is minimal.

The SAFs in Figure A6 show additional graphical representations of the energy consumption dynamics for each node at a linear scale. For each realization, the behavior was studied in terms of its falling smoothness and of his persistence with increasing delay. The M-mode was also used for this case, allowing us to simultaneously represent the SAF of the ten runs at each node. The bootstrap replication of the averaged autocorrelation per node was obtained with a 95% confidence level. This analysis revealed that the Coordinator Node has slow exponential-like and anti-persistent drop, indicating its absence of seasonality. On the other hand, Nodes 1 and 3 were fast-dropping and persistent, i.e. they were processes that follow seasonal behavior, whereas Nodes 2 and 4 were uncorrelated processes. Although their fall was slow and persistent in the initial delays, the repetitiveness was lost for larger delays. Similar to what happened in the *pdf* analysis, the coordinator and Node 4 presented wider CI of the FAS, for already explained reasons.
